# Renal Cell Carcinoma Metastasis to an Uncommon Site: The Orbital Bone

**DOI:** 10.7759/cureus.4606

**Published:** 2019-05-07

**Authors:** Zainab Shahid, Ricci Kalayanamitra, Andrew Groff, Muhammad F Khalid, Rohit Jain

**Affiliations:** 1 Internal Medicine, Lake Erie College of Osteopathic Medicine, Erie, USA; 2 Emergency Medicine, Penn State Health Milton S. Hershey Medical Center, Hershey, USA; 3 Internal Medicine, Penn State Health Milton S. Hershey Medical Center, Hershey, USA

**Keywords:** metastatic renal cell carcinoma, orbital metastasis, orbital mass

## Abstract

Renal cell carcinoma (RCC) represents 90% of all renal cancers. Patients may present with weight loss, hematuria, abdominal mass, abdominal pain, fever, and night sweats. The classic symptoms of flank pain, hematuria, and a palpable flank mass occur in less than 10% of patients and suggest advanced disease. However, most patients are typically asymptomatic and diagnosed incidentally.

RCC metastasizes most commonly to the lung parenchyma, bone, liver, and brain and less commonly to the thyroid, pancreas, muscle, skin, and soft tissue. It is very rare for RCC to metastasize to the orbital bone. We present a case of a patient who presented with left cheek pain, tingling, and numbness and was ultimately found to have orbital metastasis of RCC.

## Introduction

Renal cell carcinomas (RCCs) consist of a group of morphologically distinct adenocarcinomas which arise from the renal cortex and are classified on the basis of morphology, growth pattern, cell of origin, and histochemistry. The most common subtype of RCC is clear cell carcinoma, which constitutes 75-85% of all RCCs [1]. RCC has been found to infiltrate vasculature through the interlobular, arcuate and interlobar veins and metastasize through the systemic circulation and to the head through Batson’s venous plexus [2]. In addition, RCC promotes angiogenic factors and hence metastatic RCC tumors are highly vascularized [3]. Most cases of RCC do not cause symptoms until they are advanced; in fact, up to 40% of renal masses are found incidentally and approximately 25% of patients with RCC have metastasis at initial presentation [4]. Orbital metastases are an extremely rare complication of renal cell carcinoma and few cases of this occurrence have been reported in literature [3,5-12].

## Case presentation

An 87-year-old male with a past medical history significant for a Bosniak class 2F renal mass found incidentally in 2015 and a two-month history of pain, tingling, and numbness of the left cheek presented to the emergency department with worsening weakness and dyspnea on exertion. Two months prior to this visit, the patient was seen at an outpatient neurology clinic for numbness of the eye and lip and lancing pain to the jaw.

**Figure 1:**
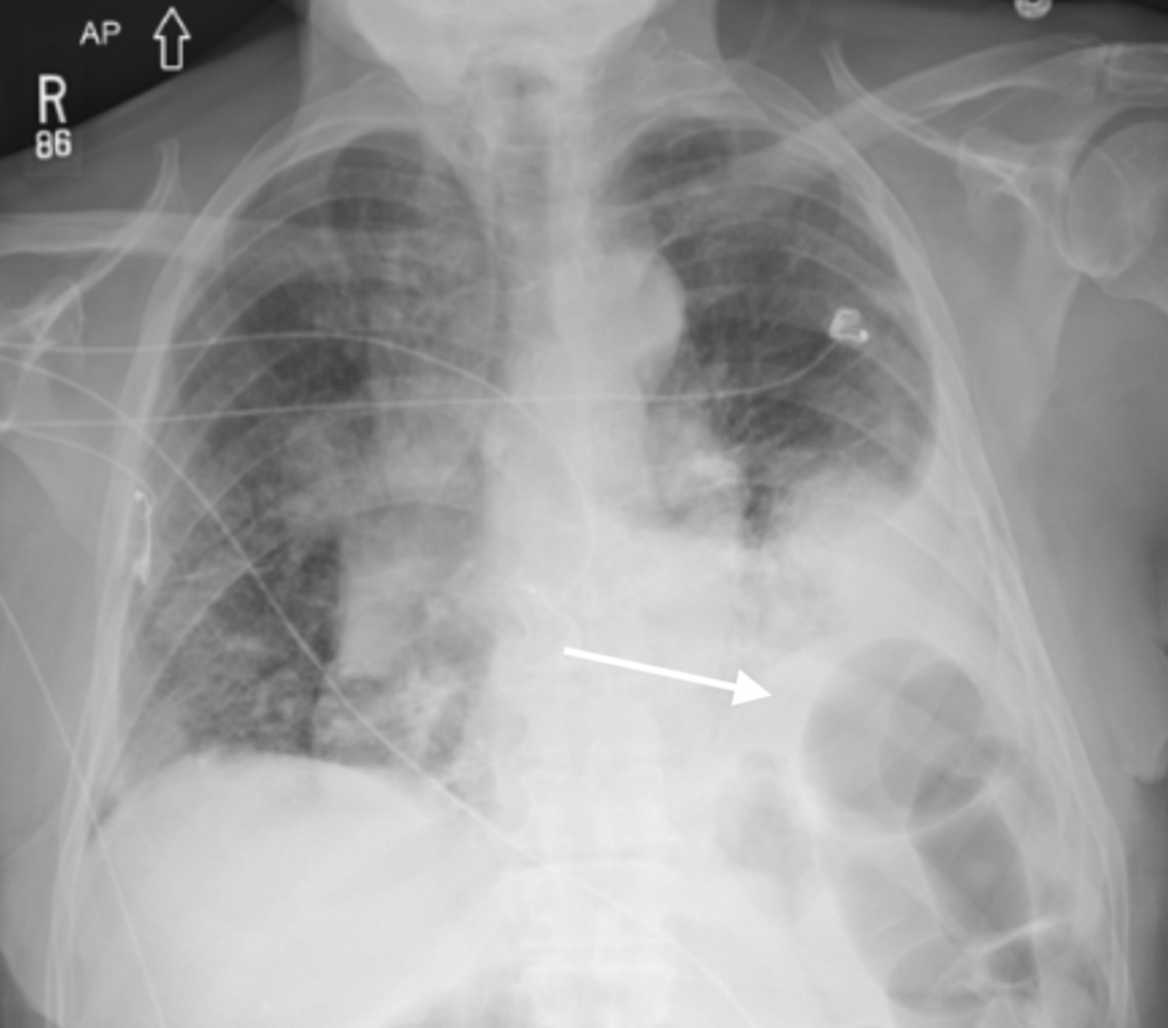
Upright chest X-ray revealing a dense airspace opacity at the left lung base (white arrow) suggestive of pleural effusion.

**Figure 2:**
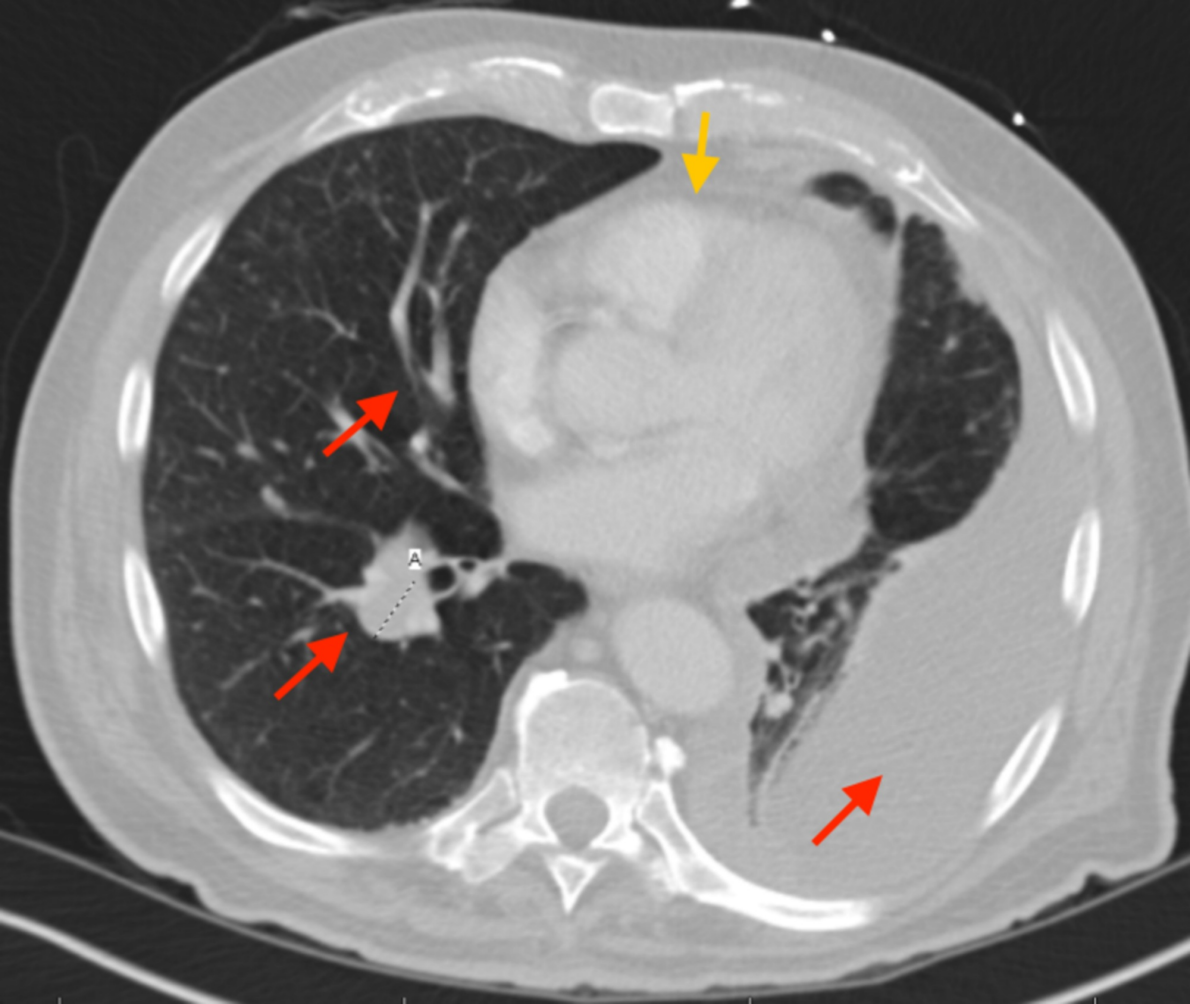
Chest CT scan with contrast revealing mediastinal adenopathy (yellow arrow) and numerous spiculated nodules of various sizes scattered throughout both lungs (red arrows). CT: Computed tomography

In the emergency department, the patient underwent a chest X-ray (Figure 1) which revealed a left-sided pleural effusion. A follow-up computed tomography (CT) scan (Figure 2) showed mediastinal adenopathy and numerous spiculated lesions in the lung which were concerning for malignancy considering the patient's history of a renal mass. A thoracentesis was performed, and the pleural fluid cytology revealed atypical cells that stained positive for PAX8 and negative for B72.3 and MOC31, consistent with cells of renal origin.

**Figure 3:**
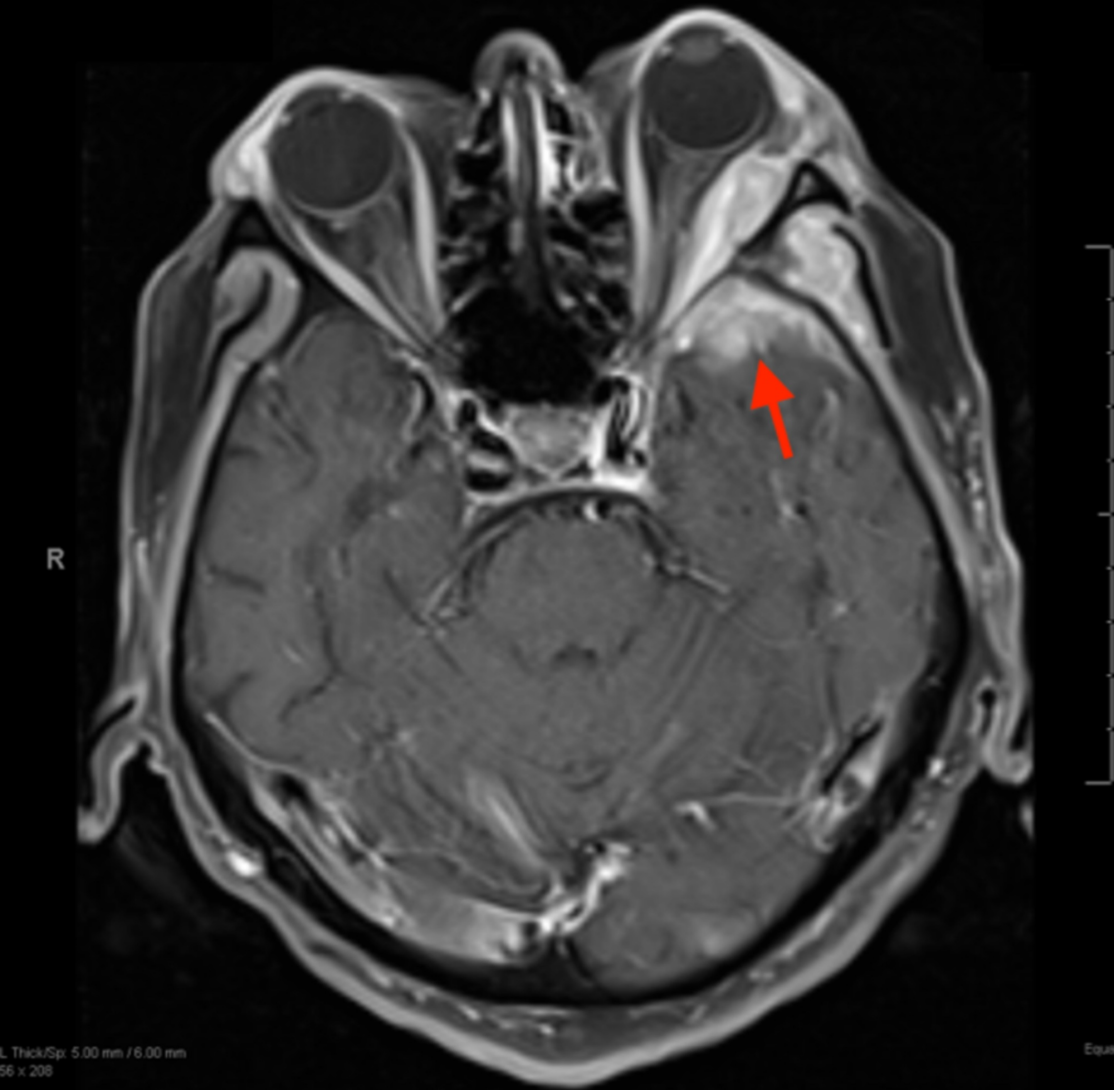
Axial T1 MRI of the brain with contrast revealing a 4.3 cm enhancing mass (red arrow) in the left anterior temporal and lateral orbital wall. MRI: Magnetic resonance imaging

**Figure 4:**
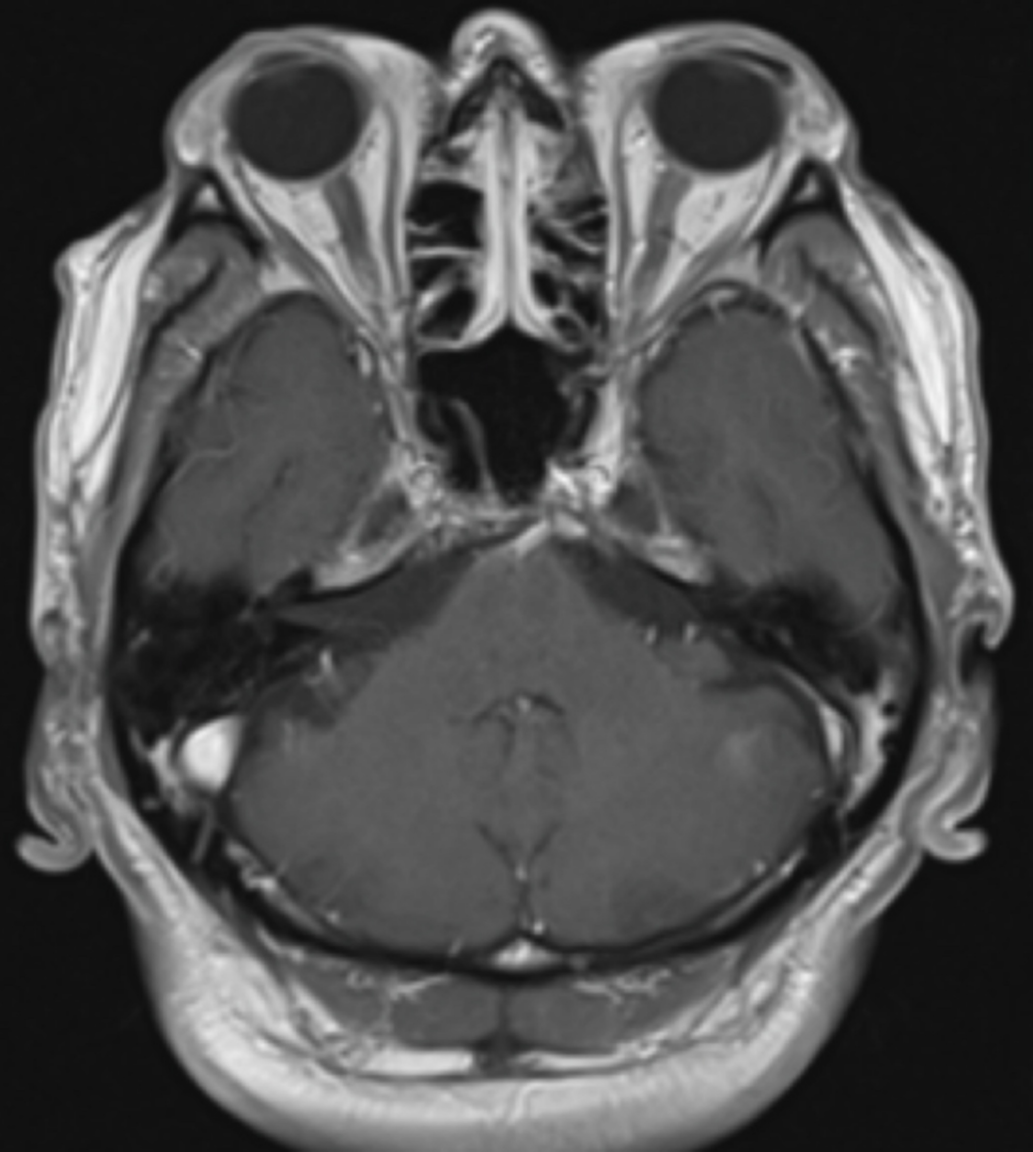
Axial T1 MRI of the brain with contrast from 2014 showing no acute intracranial abnormalities or masses. MRI: Magnetic resonance imaging

Given this finding and the patient's recent symptoms of facial pain, numbness, and tingling, the patient underwent magnetic resonance imaging (MRI) of the brain. The MRI revealed a large left anterior temporal and lateral wall enhancing mass measuring 4.3 cm (Figure 3). After comparing it with the patient’s previous orbital MRI from 2014 (Figure 4), the mass was thought to be a result of metastasis. The patient soon developed pain with extraocular movements and ultimately opted for palliative radiation.

## Discussion

Orbital metastasis of any systemic cancer is uncommon and there have been very few reports of orbital metastasis of RCC. Only two to five percent of patients with systemic cancer develop orbital metastasis and only 13% of all orbital tumors are metastatic [13]. The most common primary tumors with orbital metastases are breast, lung, and prostate. In addition, the most common clinical symptoms of metastatic orbital tumors include diplopia, proptosis, pain, decreased vision, and ptosis [14]. There have been very few previous reports of RCC metastasizing to the orbital bone.

In some reports, patients presented with orbital metastasis many years after initial diagnosis with RCC [6,8,11]. In others, symptoms due to orbital metastasis were actually the only presenting signs of RCC [4,9-13]. Radiologic workup for orbital masses can be with MRI or CT scans. Several patterns have been associated with a higher likelihood of a metastatic tumor, including intramuscular focal masses, bone destruction with a contiguous mass, and diffuse intraconal lesions, but metastatic masses may have benign characteristics on imaging as well [15]. Definitive diagnosis can be made with histopathological evaluation following fine needle aspiration, which would reveal copious clear cytoplasm, round or oval nuclei, clear cell borders, and encapsulating connective tissue [12].

Earlier diagnosis of orbital metastasis can yield a better patient prognosis by leading to a prompt diagnosis and treatment of RCC. The goal of treatment for orbital metastasis is total exenteration. Treatment of solitary orbital metastasis following radical nephrectomy of the primary tumor has been achieved with total exenteration, craniotomy, and total maxillectomy [6,7]. In one case, a patient with orbital metastasis of RCC was successfully treated with no recurrence six years after surgery and had a successful cosmetic result with an orbital prosthesis [6]. Radiation and immunotherapy have been explored as potential treatments for orbital metastasis, and chemotherapy has also been used when other treatments have failed.

## Conclusions

Although orbital metastases are rare, metastatic RCC should be considered in patients with orbital masses, even those who have an unremarkable past medical history and radiologic workup pointing towards a benign orbital mass. It is important that metastatic RCC is included in the differential diagnosis of patients presenting with atypical symptoms and an orbital mass in the settings of either RCC or a non-contributory past medical history.
